# The Programmed Cell Death of an Immature Thymocyte Cell Line Transgenic for an αβ TCR and the c-*myc* Proto-Oncogene

**DOI:** 10.1155/1995/93271

**Published:** 1995

**Authors:** Marianna Murdjeva, Yujiro Tanaka, Trisha Norton, Dimitris Kioussis

**Affiliations:** Laboratory of Molecular Immunology National Institute for Medical Research The Ridgeway London NW7 1AA United Kingdom

**Keywords:** Immature thymocytes, F5/Thy-l-*myc* transgenic mice, programmed cell death, TCR gene rearrangement, RAG-1 genes

## Abstract

The c-*myc* proto-oncogene linked to the mouse Thy-1 gene transcriptional unit predisposes mice to development of thymic tumors consisting predominantly of immature CD4^+^ CD8^+^ cells. In an attempt to immortalize immature T cells expressing a known T-cell antigen receptor (TCR), Thy-1/c-*myc* transgenic mice were bred to αβ TCR transgenic mice (F5), and CD4^+^ CD8^+^ cell lines were established from thymic tumors in double-transgenic mice. These cells expressed high-level heat-stable antigen (HSA) and were able to undergo programmed cell death upon induction with steroids and CD3 cross-linking, but not with cognate peptide. In addition, one line had rearranged and transcribed endogenous TCR c and genes, in spite of the fact that transgenic α and β genes were also expressed. Furthermore, we show that Thy-1/*myc* transgenic mice deficient in recombination activating gene-1 (RAG-1) do not develop tumors, in contrast to RAG-1^-^/^-^ mice, which are also transgenic for both Thy-1/myc and the F5 TCR. This indicates that in order for thymocytes to be transformed by the Thy-myc transgene, they need to proceed to the double-positive stage.


*Developmental Immunology*, 1996, Vol 4, pp. 279–288
Reprints available directly from the publisher
Photocopying permitted by license only

© 1996 OPA (Overseas Publishers Association)
Amsterdam B.V. Published in The Netherlands
by Harwood Academic Publishers GmbH
Printed in Singapore

### The Programmed Cell Death of an Immature Thymocyte Cell Line Transgenic for an αβ TCR and the c-*myc* Proto-Oncogene

MARIANNA MURDJEVA, YUJIRO TANAKA, TRISHA NORTON, and DIMITRIS KIOUSSIS*

*Laboratory of Molecular Immunology, National Institute for Medical Research, The Ridgeway, London NW7 1AA, UK*

The c-*myc* proto-oncogene linked to the mouse Thy-1 gene transcriptional unit predisposes mice to development of thymic tumors consisting predominantly of immature $CD4^+CD8^+$ cells. In an attempt to immortalize immature T cells expressing a known T-cell antigen receptor (TCR), Thy-1/c-*myc* transgenic mice were bred to αβ TCR transgenic mice (F5), and $CD4^+CD8^+$ cell lines were established from thymic tumors in double-transgenic mice. These cells expressed high-level heat-stable antigen (HSA) and were able to undergo programmed cell death upon induction with steroids and CD3 cross-linking, but not with cognate peptide. In addition, one line had rearranged and transcribed endogenous TCR α and β genes, in spite of the fact that transgenic α and β genes were also expressed. Furthermore, we show that Thy-1/*myc* transgenic mice deficient in recombination activating gene-1 (RAG-1) do not develop tumors, in contrast to RAG-$1^{-/-}$ mice, which are also transgenic for both Thy-1/*myc* and the F5 TCR. This indicates that in order for thymocytes to be transformed by the Thy-*myc* transgene, they need to proceed to the double-positive stage.

KEYWORDS: Immature thymocytes, F5/Thy-1-*myc* transgenic mice, programmed cell death, TCR gene rearrangement, RAG-1 genes.

### INTRODUCTION

Overexpression of the c-*myc* proto-oncogene in humans has been implicated in the development of T- and B-cell leukemia, in which chromosomal translocations bring the c-*myc* in close proximity to either TCR- or immunoglobulin-gene regulatory elements (for reviews, see Dalla-Favera et al., 1982; Casares et al., 1993; Gauwerky and Croce, 1993; Stephenson et al., 1993). In an attempt to establish immature thymocyte lines, we previously generated a transgenic mouse model carrying the murine c-*myc* under the control of regulatory sequences of T-cell-specific Thy-1 gene. Several lines of Thy-1/c-*myc* (TM) transgenic mice developed thymic tumors from which cell lines of both nonadherent (thymocytes) and adherent (epithelial cells) phenotypes were established (Spanopoulou et al., 1989). All established thymocyte lines from these mice were $CD4^+CD8^+$ double-positive and expressed high levels of transgenic c-*myc*. Analysis of TCR-gene rearrangements in the thymic tumors revealed that thymocytes were of oligoclonal origin, suggesting that oncogenic transformation occurred at the $CD4^+CD8^+$ stage in a stochastic manner. Those data supported the hypothesis that tumorigenesis is a multistep process and cooperation of c-*myc* with other genetic mutation(s) is required for neoplastic transformation, as had also been shown by others (Adams and Cory, 1992). It was postulated at the time that the secondary event necessary to cooperate with the *myc* gene and transform the thymocytes occurred at the double-positive ($CD4^+CD8^+$) stage of development.

An *in vitro* system of differentiation from double-positive to CD4 single-positive stage has been described for an immature thymocyte line (Kaye and Ellenberger, 1992). In order to generate a system that would generate CD8 single-positive cells from immortalized immature thymocytes, we bred TM mice with mice transgenic for an αβ TCR (F5) that recognizes a peptide from influenza virus nucleoprotein in the context of H-$2D^b$ (Mamalaki et al., 1992). The F5/TM double-transgenic mice

*Corresponding author.

developed thymic tumors from which we were able to derive immature $CD4^+CD8^+$ cell lines. Here we show that these double-positive (DP) cell lines are functionally immature and undergo programmed cell death upon induction with steroids and cross-linking of CD3 by antibodies. In contrast, these cell lines were refractory to treatment with antigenic peptides, suggesting either that the levels of the transgenic TCR are insufficient to mediate antigen-specific signals or that the TCP is uncoupled from CD3-linked downstream signal-transduction machineries.

In order to avoid complications of endogenous T-cell receptors interfering with selection events in the thymus and affecting the transformation processes F5/TM double-transgenic mice were bred into a Recombination Activating Gene 1 (RAG-1) deficient background (Spanopoulou et al., 1994). F5/TM/RAG-$1^{-/-}$ mice developed thymic tumors from which cell lines were isolated that expressed transiently the transgenic TCR. In contrast, TM/RAG-$1^{-/-}$ mice do not develop thymic tumors, indicating that for transformation to take place, thymocytes have to enter the double-positive ($CD4^+8^+$) stage of development.

### RESULTS

### Development of Thymic Tumors in F5.TM25 Double-Transgenic Mice

It has previously been shown that different TM mouse lines give rise to thymic tumors of various phenotypes with respect to CD4, CD8, and HSA expression (Spanopoulou et al., 1989; Y.T. unpublished observation). Thus, in an attempt to obtain thymic tumors and immortalized cell lines of immature phenotype, the TM25 line that produced HSA-$^{high}$ cell lines at a high frequency was backcrossed from (CBA × B10) $F_1$ to B10 background until they were H-$2^{bb}$ and then to F5 TCR transgenic mice. Double-transgenic mice (F5/TM$25^{bb}$) developed thymic tumors after similar latency periods to the parental TM25 mice. Peripheral lymphadenopathy was also observed in some F5/TM25 mice.

In order to determine the phenotype of cells expanded in the thymic tumor, thymic tumor cells were stained for CD4, CD8, and CD3, Vβ11(transgenic TCR), or HSA; lymphnode cells were also stained with CD4 and CD8. Figure 1 shows analysis of lymphoid tissues from a F5.TM25.1 mouse that developed a thymic tumor but did not have peripheral metastasis. The thymic tumor consisted largely of $CD4^+CD8^+$ double-positive (DP) cells (76.7%), but also contained $CD8^+$ (5.2%) and $CD4^+$ (12.4%) single-positive (SP) cells (Fig. 1a). The number of SP cells in the lymph node was similar to that of normal mice, but there was a skewing toward $CD8^+$ cells due to positive selection of the class I MHC-restricted F5 TCR transgenic T cells (Fig. 1b). Thymic tumor cells in the F5.TM25.1 mouse expressed CD3 (Fig. 1c, solid line), and Vβ11 (Fig. 1d, solid line) at levels comparable to those seen in the thymus of F5 mice (broken line). Also, F5.TM25.1 thymocytes were immature as judged by their high levels of HSA, as shown in Fig. 1e. These data suggest that immature DP thymocytes expressing the transgenic TCR were transformed in F5.TM25.1 mouse.

### Establishment of Immature DP Cell Lines from F5.TM25.1 Mouse

In an attempt to establish immature T-cell lines expressing a known TCR, F5.TM25.1 thymic tumor cells were cultured *in vitro* as described before (Spanopoulou et al., 1989). Several cell lines and clones were derived, all of which expressed high levels of CD4 and CD8. Figure 2a illustrates expression of CD4 and CD8 on a representative clone F5.TM25.1.2 (25.1.2) compared to F5 thymocytes. Expression of TCR and HSA on 25.1.2 cells were also assessed in comparison with adult F5 thymocytes. As shown in Fig. 2b, 25.1.2 cells expressed low to intermediate levels of CD3 as compared to that of DP cells in F5 mice, considering the higher auto-fluorescence (dashed line) of cultured 25.1.2 cells. In contrast, the level of Vβ11 on 25.1.2 cells was much lower than that on most F5 thymocytes (Fig. 2c) or cells freshly isolated from the tumor. Such a difference between levels of CD3 (low or intermediate) and Vβ11 (very low) suggested that 25.1.2 cells have different CD3/Vβ11 stoichiometry from that of F5 thymocytes, indicating that in the cultured tumor cells, other receptors may be expressed competing with transgenic-receptor molecules for CD3 complexes. Furthermore, higher levels of HSA expression were observed on the line compared to fresh tumor cells and this was taken as indication that the 25.1.2 cells are immature (Fig. 2d).

In order to assess the expression of transgenic and/or endogenous TCR genes in 25.1.2 cells, total

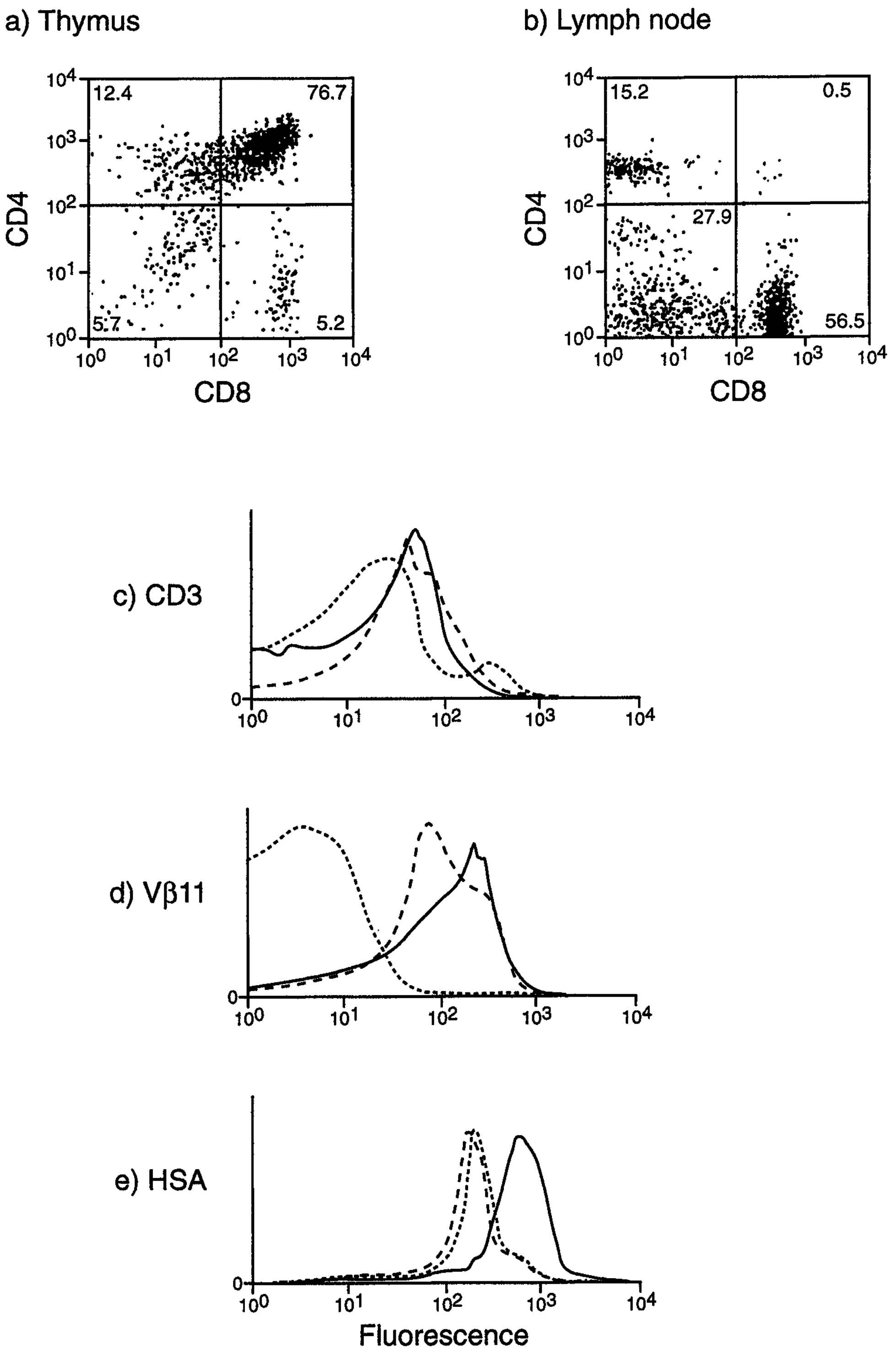

FIGURE 1. Phenotype of T cells in the thymus and periphery of F5.TM25.1 mouse. (a) Expression of CD4 and CD8 on thymic tumor cells in F5.TM25.1 mouse compared with normal F5 mouse. (b) Expression of CD4 and CD8 on lymph-node cells in F5.TM25.1 mouse. A large proportion of CD8 SP cells is characteristic of F5 transgenic mice. In this particular mouse, there was no metastasis of DP thymic tumor cells observed in some other mice. Histograms showing levels of (c) CD3, (d) Vβ11. and (e) HSA on thymic tumor cells are shown by solid lines. Dotted lines and broken lines in each histogram represent the levels of these molecules on nontransgenic and adult F5 thymocytes, respectively.

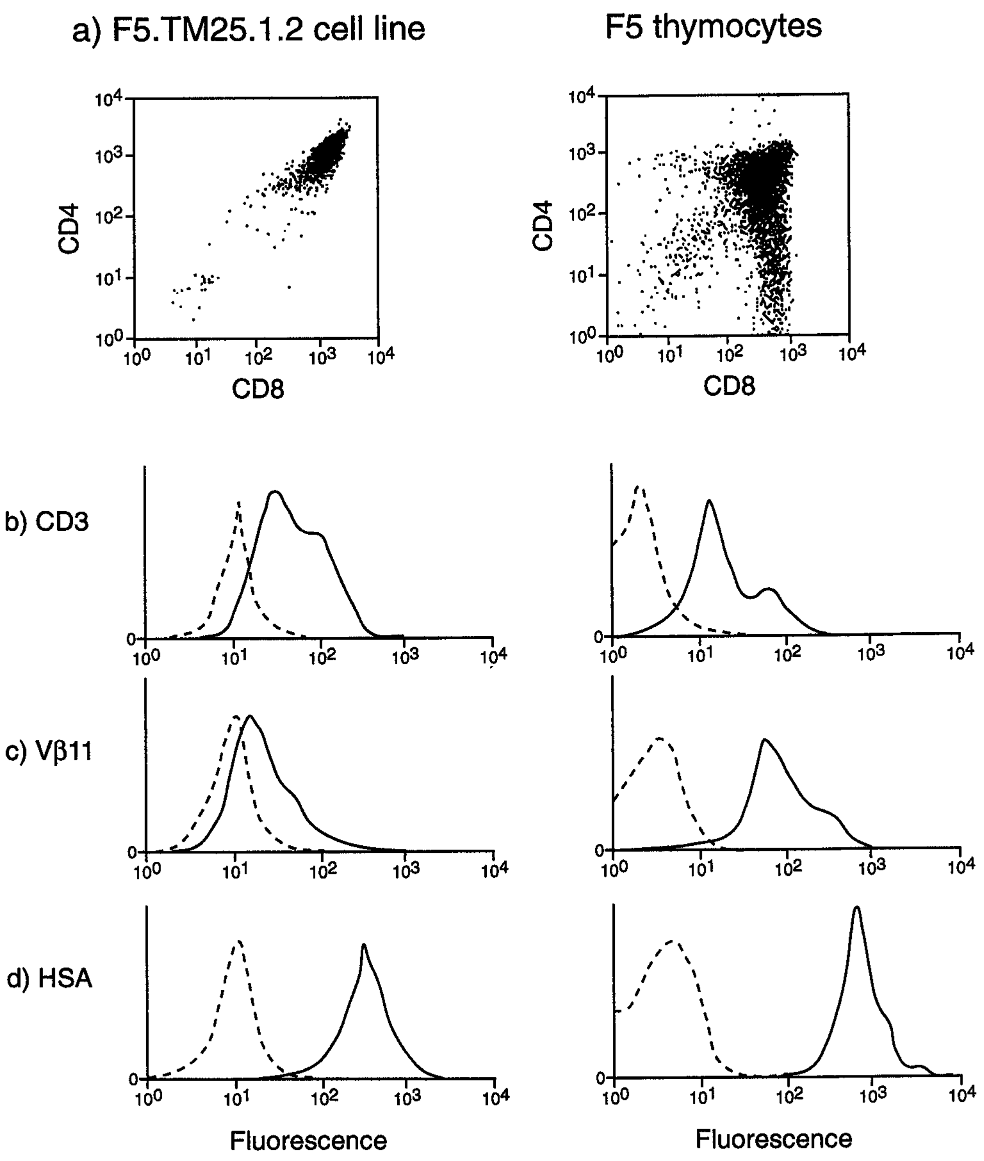

FIGURE 2. Phenotype of F5.TM25.1.2 cell line. (a) Expression of CD4 and CD8 on 25.1.2 cells and F5 thymocytes. Histograms illustrating expression of (b) CD3, (c) Vβ11, and (d) HSA. Left panels and right panels represent 25.1.2 and control F5 thymocytes, respectively. Dotted lines in histograms are of unstained controls.

RNA was extracted and reverse-transcribed to generate complementary DNA. For PCR amplification of transgenic TCR chains, sense primers for Vα4 and Vβ11 were used in combination with antisense primers for a part of the expression cassette (exon 2 of human CD2 gene located downstream of the cDNA inserts) (Mamalaki et al., 1993). As shown in Figure 3a, PCR products of predicted sizes were detected for both transgenic α and β chains, providing evidence that 25.1.2 cells transcribe the F5 TCR α and β transgenes. Next, in order to assess whether endogenous α and β TCR genes were expressed by these cells, panels of sense primers for different Vα and Vβ segments were used with antisense primers for α or β constant regions.

Figure 3b shows the presence of PCR products from Vα4 (transgenic) and Vα8 segments, demonstrating that an endogenous TCR α gene is rearranged and transcribed in 25.1.2 cells. Furthermore, 25.1.2 cells express an endogenous TCR β chain

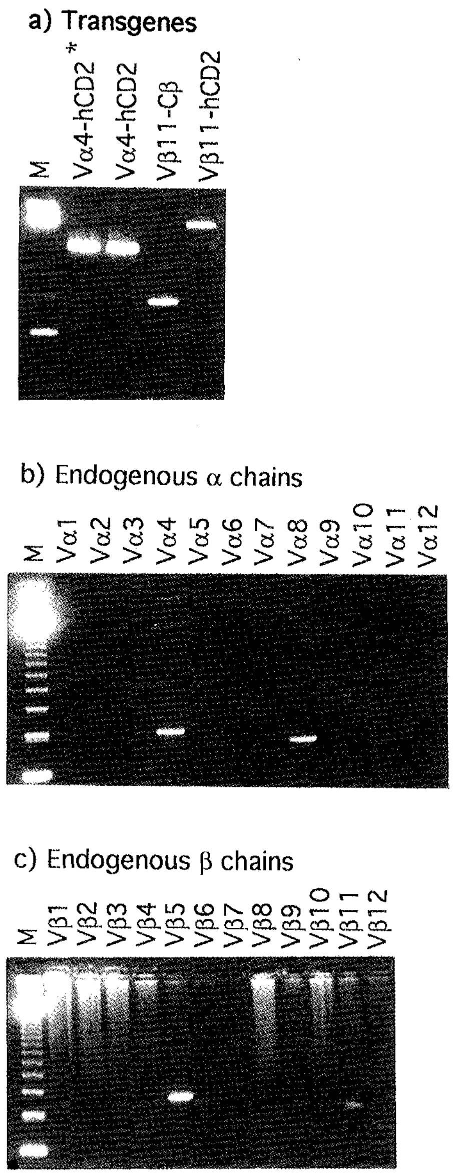

FIGURE 3. RNA-PCR analysis of TCR gene transcripts. (a) Transgenic TCR products were detected using sense primers for Vα4 and Vβ11 in combination with antisense primers for a part of the vector (hCD2) or the constant region of the β chain. mRNA from 25.1.2 cells was used, except for one sample in which the mRNA was isolated from the parental 25.1 cell line and is indicated by an asterisk (*). (b) Expression of rearranged endogenous TCR α loci was examined using a panel of sense primers for Vα1–Vα12 gene segments with an antisense primer for Cα. A Vα4 specific band could be derived either from transgenic or endogenous α chains, whereas a product for Vα8 could only originate from endogenous α gene. (c) Rearrangement of endogenous TCR β genes was assessed using a panel of Vβ1–Vβ12 family-specific sense primers and a Cβ antisense primer. A band for Vβ11 could be due to mRNA from either the transgenic or an endogenous gene. The one for Vβ5 can only come from transcription of an endogenous TCR β gene. Markers used were concatamers of 123-bp DNA fragments.

(Vβ5) in addition to transgenic Vβ11, as shown in Fig. 3c. Although the presence of transcription products from endogenous α and β TCR genes does not prove they are expressed as proteins on cell surface, the finding that endogenous chains are also expressed in these cells may explain the higher stoichiometry of CD3 over Vβ11.

### F5.TM25.1.2 Cells Undergo Programmed Cell Death

In order to examine if 25.1.2 retained characteristics of immature DP thymocytes, 25.1.2 cells were treated for 12 hr with $1 \times 10^{-6}$ M hydrocortisone, a known steroid inducer of programmed cell death in immature thymocytes (Wyllie, 1980). Total DNA was extracted after treatment and was analyzed in an agarose gel. As shown in Fig. 4a, the steroid-induced programmed cell death (apoptosis) in 25.1.2 cells produced a prominent ladder of fragmented DNA.

F5 TCR-bearing immature double-positive thymocytes respond *in vivo* or *in vitro* to cognate peptide (influenza nucleoprotein 1968 αα366–374) by undergoing apoptosis. Thus, we studied the effects of cognate peptide on the F5.TM cell line. 25.1.2 cells were cultured with peritoneal macrophages in the presence of various concentrations of the peptide recognized by the F5 TCR (1968 NP). Figure 4b shows viable cell numbers relative to those seen in cultures without peptide. It appeared that 25.1.2 cells were not affected by cognate peptide, whereas F5 thymocytes were deleted at as low as $10^{-9}$ M concentration of the peptide as reported before (Tanaka et al., 1993). CD4 or CD8 levels on 25.1.2 cells did not change after peptide treatment (data not shown). These data are in contrast to the previous observation of steroid-induced programmed cell death of 25.1.2 cells. Such a resistance of 25.1.2 cells to peptide treatment could be either due to insufficient level of TCR expression (already lower than that observed on fresh tumor cells) or a defect in TCR-linked signal-transduction pathways. Thus, if 25.1.2 cells expressed endogenous TCR β and α chains instead of transgenic F5 TCR, as discussed before (Fig. 3), 25.1.2 cells might not recognize the 1968 NP. Alternatively, if the low levels of F5 TCR present on the 25.1.2 cells were sufficient to confer antigen-specific response, there could be uncoupling of the TCR and CD3 or its downstream signal-transduction system in the cell.

In order to resolve this issue, 25.1.2 cells were stimulated by direct cross-linking of CD3ε with the antibody 145-2C11 (Leo et al., 1987) through Fc receptors on a macrophage cell line J774A.1. Coculture of 25.1.2 cells with J774A.1 cells alone did not affect 25.1.2 cells (Figure 4c). In contrast, addition of 2C11 at 70 ng/ml or 10 μg/ml concentration caused reduction in numbers of viable 25.1.2 cells, which was associated with DNA fragmentation, as shown in Fig. 4d. These data demonstrate that the 25.1.2 cell line has an intact CD3-linked signal-transduction mechanism, and that the most likely reason for their nonresponsiveness to the antigenic peptide is the low level of transgenic T-cell receptor on these cells.

### Thymocyte Lines from F5/Thy-*myc* Mice Unable to Rearrange Endogenous TCR Receptors

In order to avoid the complications of endogenous T-cell receptors being expressed during thymic development of F5/Thy-*myc* double-transgenic mice, use was made of a mouse line that is unable to rearrange endogenous T-cell-receptor genes. These mice are deficient in the Recombination Activating Gene 1 (RAG-1) (Spanopoulou et al., 1994). In RAG-$1^{-/-}$ mice, thymocyte development is arrested at the double-negative $CD4^-8^-$ stage. However,s if such mice are crossed to transgenic mice carrying an already rearranged β-chain transgene, they proceed to the double-positive ($CD4^+CD8^+$) stage (Mombaerts et al., 1992).

F5/Thy-*myc* mice in RAG-$1^{-/-}$ background were generated and these developed thymic tumors at 2 to 6 months of age. Thymic tumor cells were stained with CD4-, CD8-, Vβ11-(transgenic), and HSA-specific antibodies, analyzed on a fluorescence-activated sorter, and the results are shown in Table 1. Interesting, but without explanation at the moment, is the high incidence of metastasis to peripheral lymphoid organs of these mice as opposed to F5/TM/$RAG^{+/+}$ mice. Cell lines were isolated from these tumors and were analyzed for expression of CD4, CD8, Vβ11, and HSA. Such cells were also unresponsive to antigenic stimulus and parallel studies indicated that whereas fresh tumor cells were expressing high levels of F5 TCR, the lines showed low or undetectable levels (data not shown).

TABLE 1
FACScan Anaysis on Tumors in F5/TM/$RAG^{-/-}$ Mice

| Mouse | Age (weeks) | Thymus | | Metastasis |
|---|---|---|---|---|
| | | Vβ11 | HSA | |
| F5/TM/$RAG^{-/-}$.1 | 8 | Int. | Low | + |
| F5/TM/$RAG^{-/-}$.2 | 18 | High | Int. | + |
| F5/TM/$RAG^{-/-}$.3 | 22 | High | High | – |
| F5/TM/$RAG^{-/-}$.4 | 26 | Low | Low | + |
| F5/TM/$RAG^{-/-}$.5 | 22 | Low | Int. | + |
| F5/TM/$RAG^{-/-}$.6 | 26 | Low | Low | + |

Thymic tumors, lymph nodes, and spleens were stained for CD4, CD8, and Vβ11. Expression of Vβ11 and HSA varies amongst the thymuses. Peripheral metastasis of $CD4^+CD8^+$ thymic tumor cells was observed in lymph nodes, spleens, or both.

### RAG-$1^{-/-}$/Thy-1-*myc* Mice Do Not Develop Thymic Tumors

As mentioned before, in order for Thy-1-myc thymocytes to be transformed and immortalized, a secondary event is necessary. It has been postulated before that the secondary event probably occurs at the double-positive stage. Thy-1-*myc*/RAG-$1^{-/-}$ mice offer a unique genetic combination that could further resolve this question. Indeed, we examined over 30 Thy-1-*myc*/RAG-$1^{-/-}$ mice at ages from 3 months to 1 year and none of them showed any signs of tumors. In contrast, as described before, most of the $F5^+$/Thy-1-$myc^+$/RAG-$1^{-/-}$ mice developed thymic tumors. This observation proves that in order for Thy-*myc* thymocytes to be transformed, they have to proceed to the double-positive stage in development.

There could be two reasons why Thy-1-*myc*/RAG-$1^{-/-}$ mice do not develop tumors. It is possible that the secondary event that cooperates with the *myc* transgene for transformation occurs at the double-positive stage. Alternatively, it is possible that the *myc* transgene is not expressed in the double-negative stage at levels that can transform the thymocytes. In order to assess the latter possibility, RAG-$1^{-/-}$ and RAG-$1^{+/+}$ thymocytes were stained with Thy-1 antibodies. Figure 5 shows that in RAG-$1^{-/-}$ mice, the double-negative $CD4^-8^-$ thymocytes express Thy-1 at high levels. This indicates that the Thy-1 promoter is active in these cells, excluding the possibility of transcriptional inactivity of the Thy-*myc* transgene.

### DISCUSSION

Immature T-cell lines were established from thymic tumors in mice transgenic for Thy-1/c-*myc* and F5 TCR. The 25.1.2 cells express CD4 and CD8 at high levels and are immature as judged by high-level

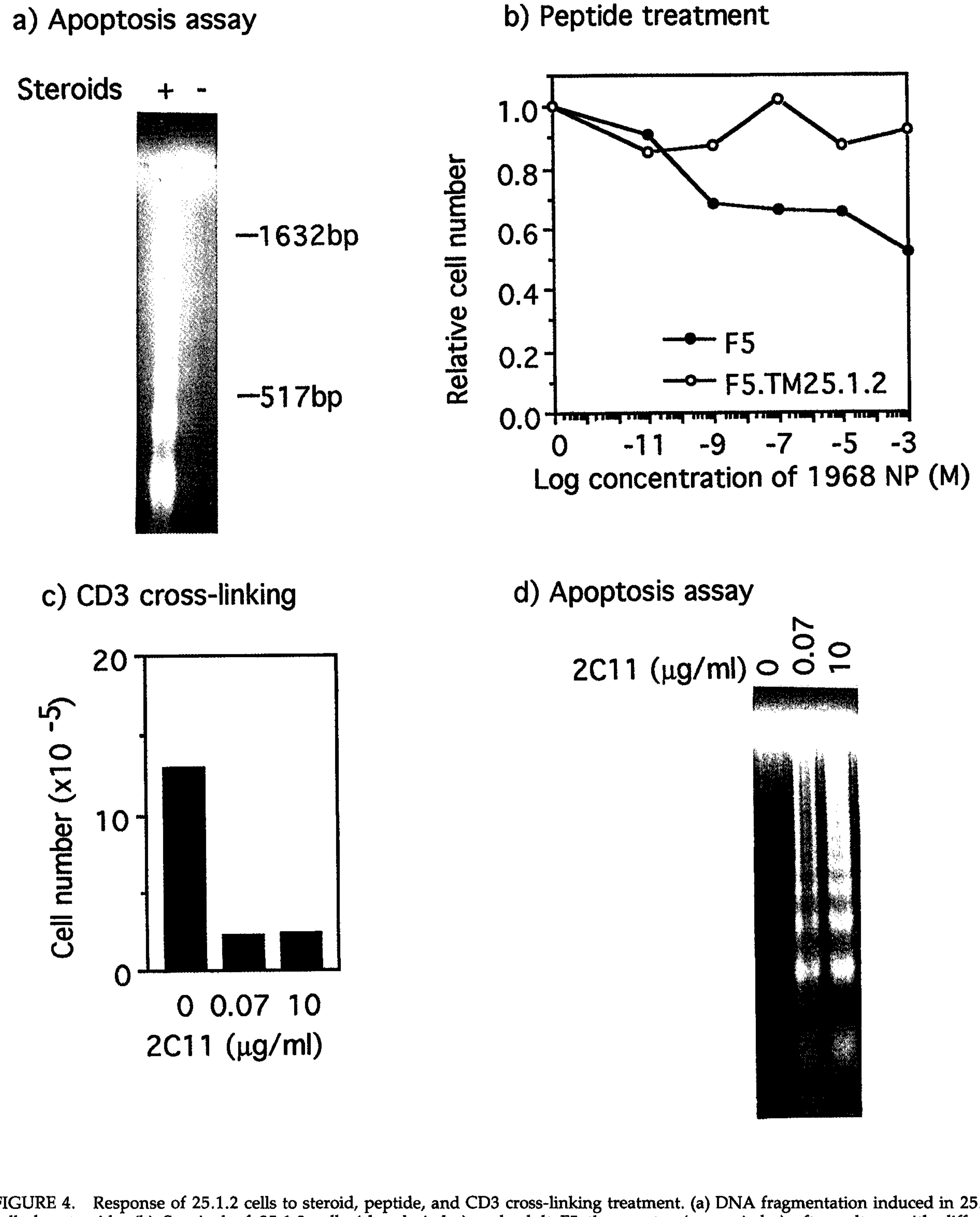

FIGURE 4. Response of 25.1.2 cells to steroid, peptide, and CD3 cross-linking treatment. (a) DNA fragmentation induced in 25.1.2 cells by steroids. (b) Survival of 25.1.2 cells (closed circles) and adult F5 thymocytes (open circles) after culture with different concentrations of 1968 nucleoprotein peptide. Whereas the 1968 NP caused deletion of F5 thymocytes at $10^{-9}$ M concentration, it did not affect 25.1.2 cells even at the highest concentration. Cross-linking of CD3 on 25.1.2 cells by antibody (2C11) bound to Fc receptors on macrophage cell line J774A.1. The anti-CD3 antibody caused deletion of (c) 25.1.2 cells by programmed cell death as shown by characteristic (d) DNA ladders at concentrations of 70 ng/ml or 10 μg/ml.

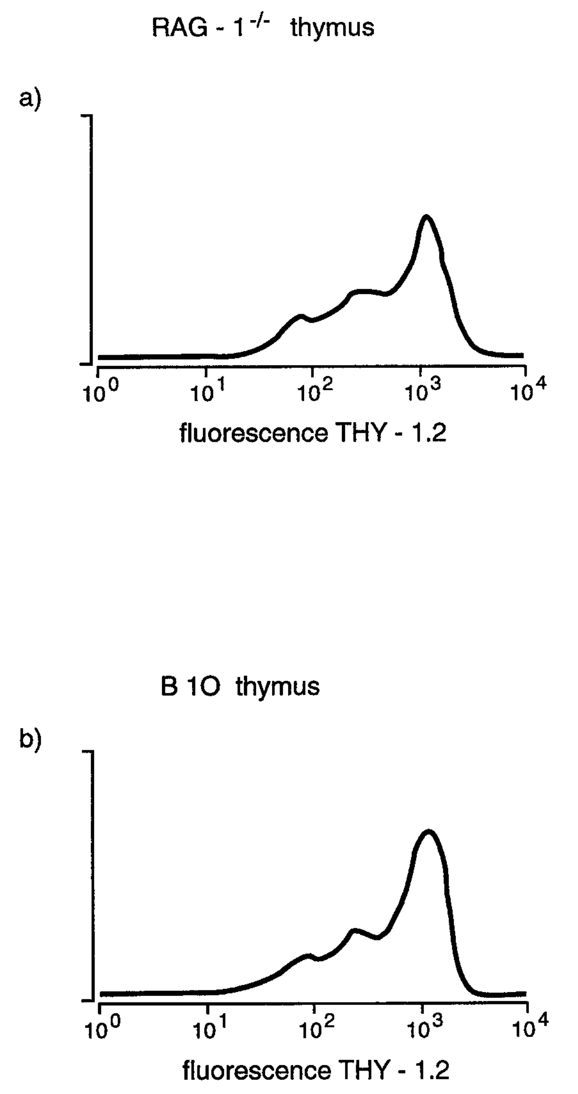

FIGURE 5. Thy-1.2 expression on RAG-1 deficient and normal (10) thymocytes. (a and b) Histograms show the staining of RAG-$1^{-/-}$ and B10 thymocytes with anti-Thy-1.2 mAb.

HSA and their ability to undergo programmed cell death upon induction with steroids. In contrast to steroid treatment, however, 25.1.2 cells did not respond to cognate peptide. This could be due to uncoupling of the TCR/CD3 complex to signaling pathways within the cell. To test this possibility, CD3 was cross-linked on those cells; this led to marked apoptosis, indicating that the CD3 complex is linked functionally to signal-transduction molecules. Therefore, the inability to induce apoptosis in these cells with antigenic peptide is probably due to insufficient levels of expression of the F5 TCR on their surface. Alternatively, these cells may represent that population of immature thymocytes that are resistant to negative selection through TCR cross-linking, because not all of their TCR molecules are coupled to the CD3 complex (Finkel et al., 1989). Thus, signaling through CD3 would be efficient in such cells and lead to apoptosis, whereas engaging the T-cell receptor might be less effective.

25.1.2 cells transcribed both transgenic α and β chains, but also contained mRNA for rearranged endogenous α and β chain genes. It is generally accepted that allelic exclusion of TCR β genes is strict and introduction of rearranged TCR β genes in transgenic mice prohibits thymocytes from rearranging their endogenous TCR β genes. The fact that mRNA for endogenous rearranged TCR genes was found in the 25.1.2 cells could be due to allelic inclusion in F5 TCR transgenic mice. In fact, the $CD4^+$ cells developing in these mice are selected exclusively on endogenous receptor chains (α and or β) (Corbella et al., 1994). Alternatively, it is possible that immortalized thymocytes continue to rearrange TCR genes (Malissen et al., 1992).

Allelic exclusion of TCR α genes, on the other hand, is less strict and expression of dual TCR α chains has been demonstrated in normal T cells (Padovan et al., 1993). Our data on expression of rearranged endogenous TCR genes in the F5 TCR transgenic 25.1.2 cell line support the hypothesis that this line is able to express, at the RNA level, two T-cell receptors. CD3 levels on these cells also suggest that possibly both receptors are expressed on the surface.

In order to exclude the complications arising from allelic inclusion or continuous rearrangement, we crossed the F5/Thy-*myc* mice to mice deficient for the RAG-1 gene. F5/Thy-*myc*/RAG-$1^{-/-}$ mice developed thymic tumors, thus excluding the possibility that endogenous receptor expression (and signaling) was necessary for transformation of thymocytes. The tumors themselves from both F5/TM/$RAG^{+/+}$ and F5/TM/$RAG^{-/-}$ mice contained cells that expressed the transgenic F5 β chain at appreciable levels. However, when placed in culture, these cells would gradually downregulate the expression of the transgenic receptor in a course of 6 months. It is not clear at the moment why this occurs. One possibility is that they lose copies of the transgene. Nevertheless, transgenic mRNA could still be detected making this an unlikely explanation.

An interesting point that arose from breeding the Thy-*myc* transgene into the RAG-$1^{-/-}$ background was that TM/RAG-$1^{-/-}$ mice did not develop thymic tumors. Given that RAG-$1^{-/-}$ thymocytes do not differentiate past the double-negative stage, this finding supports our earlier hypothesis that transformation of thymocytes in Thy-*myc* mice is a rare event and needs, in addition to the *myc* gene, a secondary event that occurs at the double-positive stage.

### MATERIALS AND METHODS

### Mice

The Thy-1/c-*myc* (Spanopoulou et al., 1989) and F5 TCR (Mamalaki et al., 1992) transgenic mice were generated in our laboratory. RAG-1 deficient mice were generous gift by Eugenia Spanopoulou (Spanopoulou et al., 1994). All mice including the C57BL/10 (B10) strain were maintained in colonies at the institute.

### Flow Cytometry

Thymic tumor cells and cell lines were stained with antibodies against CD4 (phycoerythrin-conjugated, Boehringer-Mannheim), CD8 (fluorescein isothiocyanate-conjugated YTS169.4, ATCC), Vβ11 (biotinylated KT11, a gift from Dr. Tomonari), CD3ε (biotinylated 2C11-145, ATCC), heat-stable antigen (biotinylated M1/114, ATCC), Thy-1.2 (biotinylated HO13.4, ATCC), and streptavidin-tricolor (CALTAG, California) or streptavidin phycoerythrin (Biogenesis). Samples were analyzed on FACScan (Becton Dickinson) using Lysis II Software (Becton Dickinson).

### Tissue Culture

Thymic tumors were mechanically disrupted and thymocytes were cultured in 12 well plates (Nunc) containing RPMI 1640 (Gibco) supplemented with 10% (v/v) heat-inactivated fetal calf serum (Globepharm), 0.06 g/l Penicillin, 0.1 g/l Streptomycin and 10 mM N-2 hydroxyethylpiperazine-N'-2 ethanesulfonic acid. Cells were incubated at 37°C in a humidified chamber containing 5% $CO_2$. Subsequently, a clone designated F5.TM25.1.2 (abbreviated 25.1.2) was obtained by limiting dilution at 0.5 cell/well.

For analysis of programmed cell death, 25.1.2 cells were treated with $1 \times 10^{-6}$ M hydrocortisone (Sigma) for 12 hr. Total DNA was extracted from 25.1.2 cells and was run in 2% agarose gel containing ethidium bromide as described before (Kawabe and Ochi, 1992).

Peritoneal macrophages from B10 mice were prepared according to Morikawa et al., 1993). Briefly, the mouse peritoneal cavity was washed twice with cold PBS, and adherent cells were collected after incubation for 2 hr at 37°C. Adherent cells were further treated with 100 IU/ml murine recombinant IFN-γ for 12 hr, and were incubated with different concentrations of 1968 NP peptide (366–374) (synthesized at the National Institute for Medical Research (London) for 90 min prior to coculture with 25.1.2 cells. $2 \times 10^5$ peritoneal macrophages were cocultured with $5 \times 10^5$ 25.1.2 cells for 12 hr. Lymphoid cells were recovered by gentle pipetting, viable cell numbers were counted using trypan blue dye exclusion, and cells were stained for CD4, CD8, and Vβ11. As a control, thymocytes from adult F5 transgenic mice were cocultured with macrophages as before.

A murine macrophage cell line J774A.1 (TIB 67, ATCC) was kindly provided by Dr. G. Stockinger (NIMR). 25.1.2 cells were cocultured with J774A.1 in the presence or absence of anti-CD3 antibody (2C11.145) at 0.07 or 10 μg/ml. After culture for 12 hr, fragmentation of DNA in 25.1.2 cells was analyzed as described before. In a separate experiment, 25.1.2 cells and F5 thymocytes were treated with various concentrations of anti-CD3 antibody, and cell numbers and expression of CD4, CD8, and Vβ11 were analyzed.

### RNA-PCR Analysis

Total RNA was extracted from 25.1.2 cells by the guanidinium thiocyanate method (Sambrook et al., 1989), and was reverse-transcribed (RT) by Moloney murine leukemia virus reverse transcriptase (Promega) using oligo(dT) primers (Promega). One-sixth of the RT products was amplified by polymerase chain reaction (PCR) in 1.5 mM $MgCl_2$, 60 mM KCl, 15 mM Tris-HCl (pH 8.3), 6.75% glycerol, and 2U Taq polymerase (Perkin Elmer) using thermal cycler (Hybaid). Sense primers for TCR Vα and Vβ gene segments and an antisense primer for TCR α constant region have been described before (Casanova et al., 1991). In addition, the following primers specific for transgenic F5 TCR were synthesized in our institute, Vα4: ACCAGACAAGCT-TCACCTGCCAAGATAT; Vα4': CAGTATCCCGG AGAAGGTC; Vβ11: CAAGCTCCTATAGAT-

GATTC; hCD2: TCAAAATCAGAAGGAAGCTGG; and cβ: CCTTGGGTGGAGTCACATTTC. PCR reactions were also performed without cDNA as mock controls. The PCR products were analyzed in 2% agarose gels containing ethidium bromide. A molecular size marker consisting of multimers of 123 bp DNA fragments (Pharmacia) was used.

### ACKNOWLEDGMENTS

M.M. and Y.T. were supported by a grant from the Leukemia Research Fund. The authors wish to thank Mrs. Burke for secretarial assistance and Drs. Rs. Zamoyska and B. Stockinger for reagents and scientific discussions.

*(Received March 15, 1995)*

*(Accepted May 23, 1995)*

### REFERENCES

Adams J.M., and Cory S. (1992). Oncogene co-operation in leukaemogenesis. Cancer Surv. **15**: 119–141.

Casanova J-L., Romero P., Widmann C., Kourilsky P., and Maryanski J.M. (1991). T cell receptor genes in a series of class I major histocompatibility complex—restricted cytotoxic T lymphocyte clones specific for a *Plasmodium berghei* nonapeptide: Implications for T cell allelic exclusion and antigen-specific repertoire. J. Exp. Med. **174**: 1371–1383.

Casares S., Rodriguez J.M., Martin A., and Parrado A. (1993). Rearrangement of c-*myc* and c-*abl* genes in tumour cells in Burkitt's lymphoma. J. Clin. Pathol. **46**: 778–779.

Corbella P., Moskophidis D., Spanopoulou E., Mamalaki C., Tolaini M., Itano A., Lans D., Baltimore D., Robey E., and Kioussis D. (1994). Functional committment to helper T cell lineage precedes positive selection and is independent of T cell receptor MHC specificity. Immunity **1**: 269–276.

Dalla-Favera R., Bregni M., Erikson J., Patterson D., Gallo R., and C. Croce. (1982). Human c-*myc* onc gene is located on the region of chromosome 8 that is translocated in Burkitt lymphoma cells. Proc. Natl. Acad. Sci. USA **79**: 7824–7827.

Finkel T.H., Marrack P., Kappler J.W., Kubo R.T., and Cambier J.C. (1989). αβT cell receptor and CD3 transduce different signals in immature T cells. J. Immunol. **142**: 3006–3012.

Gauwerky C.E., and Croce C.M. (1993). Chromosomal translocations in leukaemia. Semin. Cancer Biol. **4**: 333–340.

Kawabe Y., and Ochi A. (1992). Programmed cell death and extrathymic reduction of $V\beta8^+CD4^+$ T cells in mice tolerant to *Staphylococcus aureus* exterotoxin B. Nature **349**: 245–248.

Kaye J., and Ellenberger D.L. (1992). Differentiation of an immature T cell line: A model of thymic positive selection. Cell **71**: 423–435.

Leo O., Foo M., Sachs D.H., Samelson L.E., and Bluestone J.A. (1987). Identification of a monoclonal antibody specific for a murine T3 polypeptide. Proc. Natl. Acad. Sci. USA **84**: 1374–1378.

Malissen M., Trucy J., Jouvin-Marche E., Cazenave P.A., Scollay R., and Malissen B. (1992). Regulation of TCR α and β gene allelic exclusion during T-cell development. Immunology Today **13**: 315–322.

Mamalaki C., Elliott J., Norton T., Yannoutsos N., Townsend A.R., Chandler P., Simpson E., and Kioussis D. (1993). Positive and negative selection in transgenic mice expressing a T-cell receptor specific for influenza nucleoprotein and endogenous superantigen. Dev. Immunol 3: 159–174.

Mamalaki C., Norton T., Tanaka Y., Townsend A.R., Chandler Ph., Simpson E., and Kioussis D. (1992). Thymic depletion and peripheral activation of class I major histocompatibility complex-restricted T cells by soluble peptide in T cell receptor transgenic mice. Proc. Natl. Acad. Sci. USA **89**: 11342–113.

Mombaerts P., Clarke A.R., Rudnicki M.A., Iacomini J., Itohara S., Lafaille J.J., Wang L., Ichikawa Y., Jaenisch R., Hooper M.L., and Tonegawa S. (1992). Mutations in T-cell antigen genes α and β block thymocyte development at different stages. Nature **360**: 225–231.

Morikawa Y., Furotani M., Matsuura N., and Kakudo K. (1993). The role of antigen-presenting cells in the regulation of delayed-type hypersensitivity. Cell. Immunol. **152**: 200–210.

Padovan E., Casorati G., Dellabona P., Meyer S., Brockhaus M., and Lanzavecchia A. (1993). Expression of two T cell receptor alpha chains: Dual receptor T cells. Science **262**: 422–424.

Sambrook J., Fritsch E.F., and Maniatis T. (1989). Extraction of RNA with guanidinium thiocyanate followed by centrifugation in cesium chloride solutions. In: Moleculars cloning: A laboratory manual, 2d ed. Xxx and Yyy, 7.19–7.22.

Spanopoulou E., Roman A.J., Corcoran L.M., Schlissel M.S., Silver D.P., Nemazee D., Nussenzweig M.C., Shinton S.A., Hardy R.R., and Baltimore D. (1994). Functional immunoglobin transgenes guide ordered B-cell differentiation in RAG-1 deficient mice. Genes Dev. **8**: 1030–1040.

Spanopoulou E., Early A., Elliot J., Crispe N., Ladyman H., Ritter M., Watt S., Grosveld F., and Kioussis D. (1989). Complex lymphoid and epithelial thymic tumours in Thy-1-*myc* transgenic mice. Nature **342**: 185–189.

Stephenson J., Akdag R., Ozbek N., and Mufti G.J. (1993). Methylation status within exon 3 of the c-*myc* gene as a prognostic marker in myeloma and leukaemia. Leuk. Res. **17**: 291–293.

Tanaka Y., Mamalaki C., Stockinger B., and Kioussis D. (1993). *In vitro* negative selection of αβ T cell receptor transgenic thymocytes by conditionally immortalized thymic cortical epithelial cell lines and dendritic cells. Eur. J. Immunol. **23**: 2614–2621.

Wyllie A.H. (1980). Glucocorticoid-induced thymocyte apoptosis is associated with endogenous endonuclease activation. Nature **284**: 555–556.

